# Diffusion-Weighted MRI Findings of Caudate Nucleus and Putamen in Patients With Obsessive-Compulsive Disorder

**DOI:** 10.7759/cureus.17023

**Published:** 2021-08-09

**Authors:** Serpil Aglamiş, Ayşe Murat Aydın, Yesim Eroglu, Gülen Burakgazi, Murad Atmaca

**Affiliations:** 1 Department of Radiology, Firat University School of Medicine, Elazig, TUR; 2 Department of Radiology, Firat University School of Medicine, Elazig, TUR; 3 Department of Radiology, Recep Tayyip Erdogan University School of Medicine, Rize, TUR; 4 Department of Psychiatry, Firat University School of Medicine, Elazig, TUR

**Keywords:** : obsessive compulsive disorder, ocd, caudate nucleus, putamen, diffusion weighted magnetic resonance imaging

## Abstract

Objective

The purpose of this study was to establish the diffusion-weighted magnetic resonance imaging (DW-MRI) findings of the caudate nucleus and putamen in patients with obsessive-compulsive disorder (OCD) and to obtain new information on the etiopathogenesis of OCD, which is still unclear.

Methods

The study comprised 20 patients with OCDs and 20 healthy volunteers. In these cases, DW-MRI and diffusion-weighted echo-planar images (DW-EPI) at b600 and b1000 gradient values were taken and the measurements were made using the apparent diffusion coefficient (ADC) maps of each group at b600 and b1000 values from the caudate nucleus and putamen.

Results

When the DW-MRI examination in patients with OCD was compared with the control group, the mean ADC values in the caudate nucleus and putamen were not found to have statistically significantly changed. In addition, there were no significant differences regarding the right and left caudate nuclei and putamen ADC values at the b600 and b1000 in the patients with OCD or the control group.

Conclusion

There are still many unknowns about the neurobiology of OCD. When the DW-MRI examination of the patients with OCD was compared with the control group in our study, no significant difference was found between the ADC values of the caudate nucleus and putamen. Further studies are required for this present study on DW-MRI in patients with OCD to be meaningful.

## Introduction

Obsessive-compulsive disorder (OCD) is a chronic disease characterized by obsessions and compulsions with a serious negative impact on a person's social and professional functionality. Obsessions are persistent, repetitive, unwanted and intrusive thoughts, images, or impulses that cause anxiety. Compulsions are repetitive behaviors or mental acts made in order to reduce the anxiety formed by these disturbing thoughts or to be protected from or to avoid feared consequences [1]. Significant differences were observed between patients and healthy controls in neuroimaging studies in OCD dating back to the 1980s [2].

The etiopathogenesis of OCD is still unclear. Brain imaging studies and neuropsychological findings suggest that various regions of the brain are involved in the development of OCD. The orbitofrontal cortex, anterior cingulate cortex, basal ganglia, and thalamus are frequently thought to be affected in OCD [3]. Using the single-photon emission computed tomography (SPECT) method, changes in blood flow have been observed in the thalamus, frontal region, and basal ganglia, compared to the control group [4,5]. In another study, increased glucose metabolism was detected in the basal ganglia and orbitofrontal cortex [6]. Likewise, different results were obtained in studies on the volumes of caudate nucleus and putamen. The volume of the caudate nucleus was found to be increased in one study, while no difference was found in the volumes of caudate nucleus and putamen between the patients with and without OCD in another study [7,8].

Diffusion-weighted imaging (DWI) is a part of routine magnetic resonance imaging (MRI). It is a technique based on the measurement of accelerated or limited diffusion movements of the protons in the water molecules in the tissue [9]. The determination of diffusion movements of water molecules provides diagnostic information about many different conditions and diseases [10,11]. In OCD, encoding the changes in the brain is very important for its diagnosis and treatment. In the present study, we compared the apparent diffusion coefficient (ADC) in the caudate nucleus and putamen of patients with OCD and healthy individuals, using the diffusion-weighted magnetic resonance imaging (DW-MRI) technique. By encoding the DW-MRI findings in the defined regions of the brain in patients with OCD, we have tried to contribute to the body of knowledge surrounding the still unknown etiopathogenesis and biological aspects of OCD. This study is the first neuroimaging study in patients with OCD using the DW-MRI technique. 

## Materials and methods

Twenty patients who were diagnosed with OCD according to Diagnostic and Statistical Manual of Mental Disorders-Fourth Edition Text Revision DSM-IV-TR and had undergone no previous drug treatment and 20 healthy volunteers, matched with the patient group in terms of age and gender, were included in the study. Patients who had another DSM-IV Axis-I disorder, a history of neurological disease or treatment, head trauma, a history of alcohol and substance addiction in the previous six months, severe systemic disease (diabetes mellitus, chronic renal disease, and hypertension), and mental retardation and individuals with an education level that might cause communication problems, were excluded from the study.

MRI study

The study was conducted using the 1.5T GE Signa High-Speed ​​scanner Excite MRI system (General Electric, Milwaukee, WI, USA). All patients were prepared for the examination in the supine position, by being centered on the head coil. No sedation was given to patients during shooting. After taking the three-plane-localizer (pilot) images, axial fluid-attenuated inversion recovery (FLAIR), T1, and coronal spin-echo (SE) T2-weighted images were obtained. As ADC values are more significant in high b values, b600 and b1000 values were studied. The b100 values were excluded from the study due to optimal ADC values ​not being obtained in lower b values and physiological events being monitored in higher b values. The parameters used in the images were:

Diffusion-weighted imaging; matrix: 128 x 128, number of excitations (NEX): 1, field of view (FOV): 36 x 36 cm, slice thickness: 5 mm, gaps between slices: 0, diffusion direction: all directions, repetition time (TR): 8,000 msn, echo time (TE): minimum.

FLAIR; matrix: 128 x 128, NEX: 1.0, FOV: 22 x 22 cm, slice thickness: 5 mm, TR: 8,000 msn, TE: 95.

T1A; matrix: 128 x 128, NEX: 1.0, FOV: 22 x 22 cm, slice thickness: 5 mm, TR: 532 msn, TE: 15.

T2A; matrix: 128 x 128, NEX: 1.0, FOV: 22 x 22 cm, slice thickness: 5 mm, TR: 4,100 msn, TE: 102.

Assessments were made of whether there was pathology in brain parenchyma with FLAIR, T1- and T2-weighted images. The diffusion-weighted images obtained were processed on a workstation of the system of MR (Advantage Windows, version 4.2 software, GE Medical Systems) and colored ADC maps of brain parenchyma were obtained. The measurements were taken from ADC maps for both b values by placing the standardized region of interest (ROI) in the caudate nucleus and putamen. The circular study area of ROIs was standardized as 30-45 mm², and their mean ADC values and standard deviations (SDs) were calculated. ADC values were calculated automatically by repeating for each b value in the unit of sec/mm².

Statistical analysis

Statistical analysis was performed with the statistical package for the social sciences (SPSS, Inc., Chicago, IL, USA) 15.0 for Windows program. The b600 and b1000 values calculated in the caudate nucleus and putamen for each patient were entered into the SPSS program separately for each group. Data were presented as mean ± standard deviation. Statistical analysis was calculated by using Student's T-test (independent two-sample). In all analyses, p<0.05 was considered to be a statistically significant result.

## Results

For a total of 40 cases, 20 patients with OCD and 20 controls were included in the study. No significant difference in age, gender distribution, and education level was observed between the groups. The mean ages of the patients with OCD and the control group were 29.4 ± 6.1 and 30.8 ± 4.2, respectively. Eighteen and 20 patients were right-hand dominant in the OCD and control groups, respectively.

**Figure 1:**
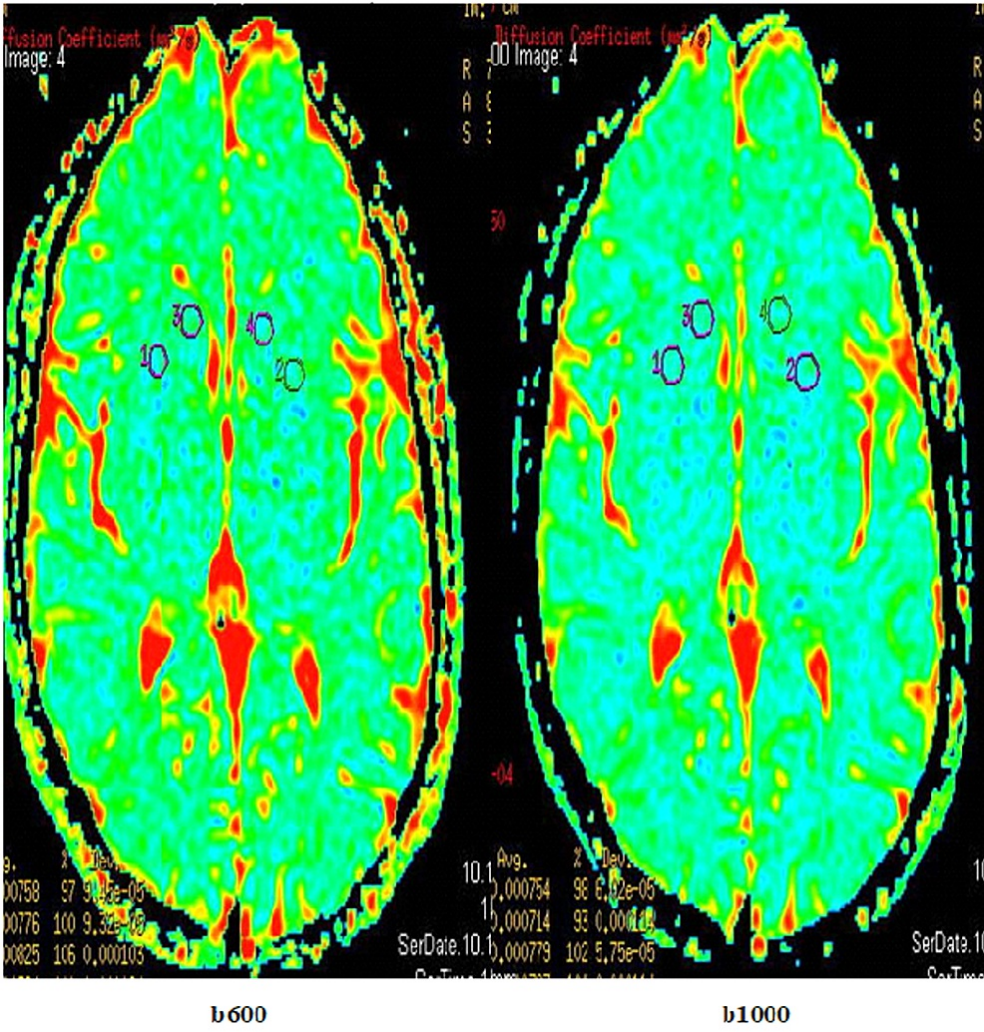
ADC maps in healthy subjects in DW-MRI taken respectively with b600 and b1000 values. ADC: apparent diffusion coefficient; DW-MRI: diffusion-weighted magnetic resonance imaging; ROI: region of interest; 1, 2, 3, and 4: ROIs used for the ADC measurement of the caudate nucleus and putamen on ADC map in subjects.

**Figure 2:**
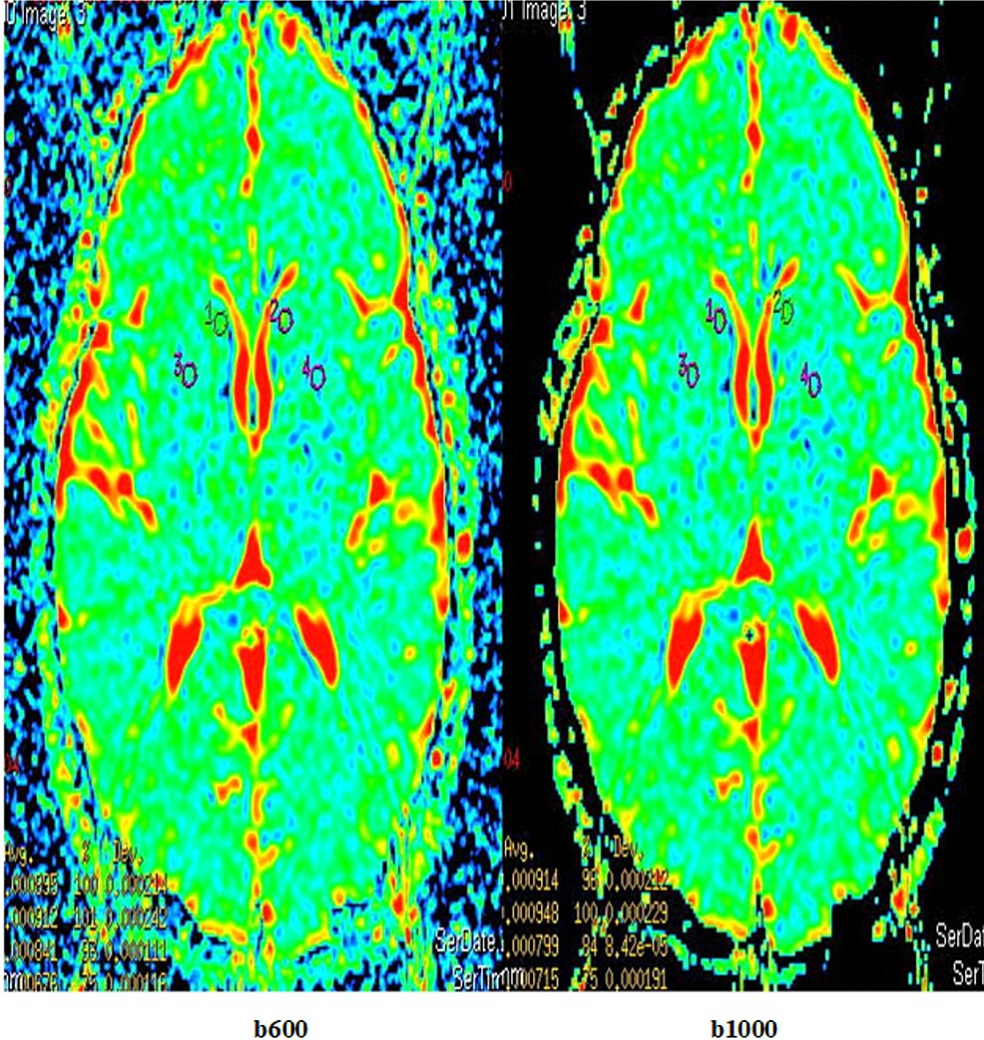
ADC maps in patients with OCD in DW-MRI taken respectively with b600 and b1000 values. ADC: apparent diffusion coefficient; OCD: obsessive-compulsive disorder; DW-MRI: diffusion-weighted magnetic resonance imaging; ROI: region of interest; 1, 2, 3, and 4: ROIs used for the ADC measurement of the caudate nucleus and putamen on ADC map in subjects.

The ADC values of the caudate nucleus and putamen of the patients with OCD and the control group were measured in sections conducted with the b600 and b1000 values on MRI (Figures 1, 2).

The left caudate nucleus ADC values were found to be higher in both the OCD and control groups in b600 and b1000 compared to the right caudate nucleus ADC values; however, the difference was statistically insignificant (p >0.05) (Table 1). No significant difference was found between the patients with OCD and the control group in the right and left caudate nuclei ADC values (p>0.05) (Table 1 ). The mean ADC values of the caudate nucleus in the OCD group were found to be lower compared to the control group in b600 and b1000 (Table 2). However, the difference was statistically insignificant (p>0.05).

**Table 1:** The ADC values of caudate nucleus (CN) in OCD and the control (C) groups (x10−6 sec/mm²) (mean values ± SD). ADC: apparent diffusion coefficient; OCD: obsessive-compulsive disorder.

	Right CN(OCD)	Right CN(C)	Left CN(OCD)	Left CN(C)
b600	827 ± 83	851.6 ± 55	855 ± 105	851.9 ± 58
b1000	736 ± 96	772 ± 52	759 ± 111	774 ± 54

**Table 2:** The mean ADC values of caudate nucleus (CN) and putamen (P) in OCD and the control (C) groups (x10−6 sec/mm²) (mean values ± SD). ADC: apparent diffusion coefficient; OCD: obsessive-compulsive disorder.

	CN(OCD)	P(OCD)	CN(C)	P(C)
b600	841 ± 80	781 ± 34	851.7 ± 101	794 ± 32
b1000	747 ± 94	696 ± 62	773 ± 47	720 ± 34

In cases of OCD, the ADC values of the right putamen were higher than the ADC values of the left putamen in b600 and b1000 (Table 3). In the comparison of the two groups, the ADC values of the right and left putamen in b600 and b1000 were found to be lower in the patients with OCD than the control group (Table 3). The mean ADC values of the putamen in the patients with OCD in b600 and b1000 were found to be lower than the values of the control group (Table 2). However, the differences were statistically insignificant (p>0.05).

**Table 3:** The ADC values of putamen (P) in OCD and the control (C) groups (x10−6 sec/mm²) (mean values ± SD). ADC: apparent diffusion coefficient; OCD: obsessive-compulsive disorder.

	Right P(OCD)	Right P(C)	Left P(OCD)	Left P(C)
b600	788 ± 45	793 ± 49	774 ± 43	796 ± 45
b1000	703 ± 68	725 ± 45	690 ± 65	718 ± 42

## Discussion

Images of the brain have been commonly used to clarify the etiopathogeneses of psychiatric disorders. Currently, the neurobiology of OCD remains unclear. The aim of this study was to contribute to the encoding of the etiopathogenesis of OCD by establishing the DW-MRI findings of the caudate nucleus and putamen.

OCD is a chronic disease rarely showing spontaneous recovery, characterized by the inability to prevent unwanted, intrusive, and repetitive thoughts. The obsessions and compulsions are time-consuming and significantly affect the person's normal routine, occupational functioning, social activities, and relationships [8].

Birth trauma, head trauma, epileptic disorders, Parkinson's disease, Huntington's disease, Sydenham's chorea, progressive supranuclear palsy, Gilles de la Tourette syndrome, frontal lobe tumors, neuroacanthocytosis, hypoxia occurred in the neonatal period, bilateral caudate infarcts, and carbon monoxide and manganese poisoning may cause OCD [12,13]. It is noteworthy that there are some lesions in various parts of basal ganglia such as the caudate nucleus, putamen, and globus pallidus in all the cases mentioned that provide very important clues, causing the basal ganglia to be prominent in the etiology of the disease [14].

Numerous studies have been carried out to elucidate the etiopathogenesis of OCD. In our literature review, no study could be found on DW-MRI in patients with OCD, with a majority comprising volumetric MRI studies.

DW-MRI provides qualitative information on various pathological changes in the brain; ADC maps enable quantitative measurement of the diffusion of water molecules, which varies in pathological conditions in the brain, in which ADC is a measure of mobility at the molecular level [15].

Basal ganglia is a widely researched area in studies on the pathophysiology of OCD. The left caudate is greater than the right caudate by volume and this is considered to be normal asymmetry [16]. As Scarone et al. showed in MRIs of patients with OCD that the volume of the right caudate head increased compared to controls and the normal asymmetry disappeared [17]. Similarly, Jenike et al. also observed a loss in asymmetry, in addition to total cerebral and cerebellar white matter reduction, in the patient group compared to controls, and an increase in total cerebral cortical volume [18].

There are also some studies using MRI that evaluate the intensity of basal ganglia in patients with OCD. While the caudate nucleus intensities were reported to be similar on T1-weighted images in the control group and the group with OCD in one study [19], caudate nucleus density was reported to be asymmetric on T1-weighted images in the group with OCD in another study [20].

In our study, there was no significant difference in the findings obtained between the ADC values of the right and left caudate nuclei both in patients with OCD and in the control group. Again, in our study, there was no significant difference between the ADC values of the right and left putamen both in patients with OCD and in the control group.

In their MR spectroscopy study with pediatric age group, Yalçın et al. determined important metabolic differences between patients with OCD and a healthy control group in the inferior frontal gyrus, anterior cingulate cortex, putamen, and globus pallidus, which are important in the etiology of OCD [21]. In this study, it was observed that choline (Cho)/creatine (Cr) and N-acetylaspartate (NAA)/Cr ratios tended to be higher in the left putamen and the globus pallidus in the OCD group. Ohara et al. were unable to detect any metabolic differences in the putamen and the globus pallidus in adults with OCD with magnetic resonance spectroscopy (MRS) [22].

In our study, there were no significant differences in the comparison of the ADC values of caudate nucleus and putamen between the patients with OCD and the control group.

The most important limitation of this present study was the low sample number. This study was supported as a Scientific Research Project by our University, so support will be limited.

## Conclusions

There are still many unknowns about the neurobiology of OCD. When the DW-MRI examination of the patients with OCD was compared with the control group in our study, a significant difference was not found between ADC values of the caudate nucleus and putamen. For this study’s findings to be meaningful, they should be supported by prospective multi-center studies.
